# Facultative polyandry under heat stress and the evolutionary potential for climate-driven shifts in mating systems

**DOI:** 10.1038/s41437-025-00795-5

**Published:** 2025-09-18

**Authors:** Matilda Q. R. Pembury Smith, Laura Latkova, Rhonda R. Snook

**Affiliations:** 1 Department of Zoology, Stockholm University, Stockholm, Sweden; 2 Bolin Centre for Climate Research, Stockholm University, Stockholm, Sweden

**Keywords:** Behavioural ecology, Sexual selection

## Abstract

The ecology of mating interactions determines a species’ mating system, yet whether environmental change can alter the mating system of a species remains unclear. Elevated temperatures can cause male sterility, prompting females to remate for fertility assurance. In monandrous systems, heat-induced male infertility poses a significant extinction risk, as females may mate exclusively with infertile males. A key question is whether male sterility could drive polyandry in a typically monandrous system. Here we address this by examining genetic variance underlying both male fertility resilience to heat stress and facultative polyandry, and assessing the fitness consequences of each mating system. We used isofemales lines of *Drosophila subobscura*, a monandrous species, exposing males to developmental heat stress. Male heat stress generated sterility and females mated to these males typically remated. While significant genetic variation in male fertility sensitivity and female remating emerged at moderate to high temperatures, we found little genetic variation in plasticity for polyandry. These results indicate evolutionary potential in both traits, but that a shift in mating system would arise through selection on genes associated with polyandry, rather than plasticity. Polyandry improved offspring production after initially mating to a sterile male, but did not fully restore reproductive output relative to fertile monandrous pairs, and mating with heat-stressed males increased female mortality. Heat stress also altered mating behaviour which could impact female mate choice. Together, these findings show that increasing temperatures may shape species’ mating systems and the interplay between thermal ecology and sexual selection under climate change.

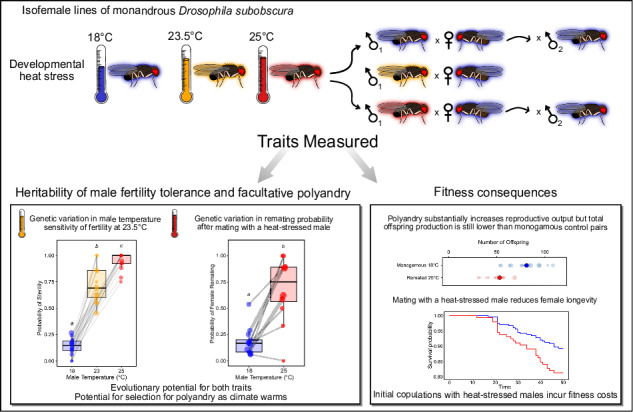

## Introduction

Animal mating systems are typically defined by the number of mates acquired by individuals of each sex over a given period and are shaped by the ecological context in which mating occurs (Emlen and Oring 1977). Abiotic change can alter the costs and benefits of mating (Candolin and Heuschele 2008; Pilakouta and Ålund 2021), leading to sex-specific shifts in reproductive strategies. When these shifts are underpinned by heritable variation, they can drive evolutionary changes in a species’ mating system (Taylor et al. 2014; García-Roa et al. 2020; Fisher et al. 2021; Leith et al. 2022). As mating systems influence the opportunity and strength of sexual selection and sexual conflict (Kvarnemo and Simmons 2013; Perry and Rowe 2018), with potential consequences for population viability (Holman and Kokko 2013), evaluating how abiotic change alters mating behaviour is essential for predicting population trajectories.

One abiotic factor that could influence mating systems is temperature. Temperature affects a broad range of biological processes (Kingsolver 2009; García-Roa et al. 2020), particularly in ectotherms whose physiology is directly affected by external temperature (Kingsolver 2009). Spermatogenesis starts in the juvenile stage for many insects (Roosen-Runge, 1977; Dumser 1980; Nijhout 1998), such that developmental heat stress can result in male infertility or sub-fertility (e.g., David et al. 2005; Sales et al. 2018, 2021; Green et al. 2019; Rodrigues et al. 2022; Canal Domenech and Fricke 2023). Male sterility can generate a cryptically female-biased operational sex ratio (Walsh et al. 2021), altering the reproductive strategy of the female to maintain fertility (Hasson and Stone 2009). For example, heat-induced male infertility increases female remating compared to control females in the polyandrous species *Tribolium castaneum* (Vasudeva et al. 2021) and *Drosophila pseudoobscura* (Sutter et al. 2019) as a fertility assurance mechanism.

In monandrous species, the cost of male sterility to population viability is likely to be greater due to the high risk of exclusively pairing with an infertile male. Whether male sterility could drive an evolutionary shift from monandry to facultative polyandry remains an open question and requires heritable variation in female willingness to remate. Polyandry generally can provide females with a fitness benefit by ensuring fertility (e.g., Price et al. 2008; Sutter et al. 2019; Vasudeva et al. 2021), but can also impose costs (Arnqvist and Nilsson 2000; Holman and Kokko 2013) such as reduced longevity (e.g., Arnqvist and Nilsson 2000; Londoño-Nieto et al. 2023) and increased sexual conflict (Holman and Kokko 2013). To understand the potential and consequences of a mating system shift under a warming climate, such as in a typically monandrous species, assessing the extent, genetic basis, and fitness effects of facultative polyandry is necessary.

Following heat stress, selection for male fertility tolerance will increase given these males will gain a reproductive advantage under hotter conditions, a selection pressure further amplified by female-biased skews in the operational sex ratio (Leith et al. 2022). A response to such selection pressures requires heritable genetic variation for male fertility tolerance. Isofemale lines of *D. melanogaster* have been shown to vary in fertility tolerance (Zwoinska et al. 2020; Rodrigues et al. 2021), with “high” and “low” tolerance lines associated with variation in candidate genes linked to fertility in *D. melanogaster* and other species (Rodrigues et al. 2022). While some broad-sense heritability was observed at high temperatures, additive genetic variance was limited (Zwoinska et al. 2020), which may constrain evolutionary responses. These contrasting findings highlight a key research gap in understanding the evolutionary potential of this trait. Additionally, if facultative polyandry was genetically associated with male fertility susceptibility, then we might expect that isofemale lines in which males had reduced fertility tolerance would have females with increased facultative polyandry. However, this relationship has not been tested.

Here, we quantify genetic variation in both heat-induced male sterility and facultative polyandry, and whether these traits appear genetically linked using the typically monogamous species, *Drosophila subobscura*. This species is a model for studying phenotypic and genetic variation in response to temperature. Independently replicated latitude-dependent chromosomal inversion frequencies have shifted in response to ambient temperature (Balanyá et al. 2006; Rezende et al. 2010). Additionally, latitudinal gene expression clines support trade-offs between metabolic and reproductive investment (Porcelli et al. 2016), and geographical variation in transcriptome plasticity following thermal selection is known (Antunes et al. 2024). Moreover, populations vary in both survival heat tolerance (Castañeda et al. 2019) and in heat-induced sterility (Porcelli et al. 2017). Though typically monandrous, one study found some *D. subobscura* females will remate when either the male or female of the interacting pair are kept at high temperatures as adults (Fisher et al. 2013). To better understand genetic variation underlying both male fertility tolerance and facultative polyandry in response to heat-induced sterility, we experimentally elevate the number of infertile males by exposing them during development to sterility causing temperatures (Porcelli et al. 2017). We use isofemale lines developing under different temperatures to assess genetic variation in these traits and their plasticity, along with the association between male sterility and facultative polyandry. In addition, offspring production and female lifespan under different mating systems were measured as selection for polyandry will be dependent on the costs and benefits of remating (Arnqvist and Nilsson 2000; Holman and Kokko 2013). While these traits were our main focus, we also examined how male heat stress affected male mating behaviour, including mating probability, mating latency and copulation duration, traits important for male and female fitness (Taylor et al. 2008; Lizé et al. 2014).

## Methods

### Stocks

Fourteen D. subobscura isofemale lines derived from wild-caught individuals from the United Kingdom (between 55°55'13.1“N 3°11'32.0“W and 55°55'56.3“N 3°11'42.4“W) were used for this study (DG −1, −2, −3, −4, −7, −8 and BF −1, −3, −4, −7, −8, −11, −12, −14). Lines were housed at 18 °C in standard culture vials containing 5 ml of a standard food medium (1 L water: 80 g medium cornmeal, 18 g dried yeast, 10 g soya flour, 80 g malt extract, 40 g molasses, 8 g agar, 25 mL of 10% Nipagin, 4 mL of propionic acid) under a 12:12 h light: dark cycle. These stocks were used to generate experimental animals. Individuals were collected in October 2021, and isofemale lines were kept for ca. 22 generations before experimental use. No ethical approval was required for the work.

### Production of focal individuals

**Fig. 1:**
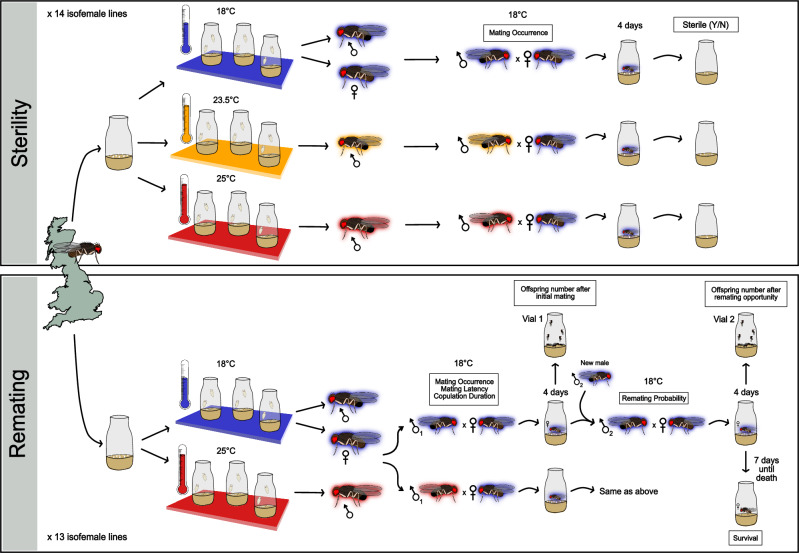
A schematic of the experimental design. Wild-caught *Drosophila subobscura* from the United Kingdom established 14 isofemale lines. Each line laid eggs for 3 days or until ca. 30 eggs were present per vial. Two experiments were conducted on separate fly sets. The first examined how male development temperature affected sterility probability and its underlying genetic variation using all 14 lines. Vials were maintained at 18 °C, 23.5 °C or 25 °C throughout development until eclosion. After eclosion, a sexually mature virgin male (developed at 18 °C, 23.5 °C or 25 °C) and female (18 °C) from the same line were paired and observed for 3 h. Mated females were left to lay eggs for 4 days. Larvae presence was checked 8 days after mating to assess male sterility. The second experiment focused on the effect of male development temperature on female remating probability and its underlying genetic variation using 13 lines. Additional behavioural and fitness measurements were taken: heat stress effects on male mating probability, mating latency and copulation duration for the first mating, offspring production and female survival. Vials were maintained at 18 °C or 25 °C throughout development until eclosion. After eclosion, a sexually mature virgin male (18 °C or 25 °C) and female (18 °C) from the same line were paired (vial 1) and observed for 3 h. Mated females were left to lay eggs for 4 days in vial 1, then were transferred to vial 2 with a new control male and observed again for 3 h. Regardless of remating status, males were removed and females were left to lay eggs for another 4 days. Afterwards, females were moved to a new vial and monitored for survival, with transfers every 7 days. Offspring from vial 1 and vial 2 were counted over 11 days from the onset of eclosion (ca. 23 days after mating). Measurements recorded during the experiment are shown in labelled boxes at relevant points in the figure.

See Fig. 1 for our experimental design. Twenty individuals of the same line (ca. 1:1 sex ratio) were placed in a vial. Parent flies were removed after 3 days or until ca. 30 eggs were laid per vial. Vials were maintained at 18 °C, a non-thermally stressful control temperature (Castañeda et al. 2013), or 25 °C, a temperature within this species’ thermal range (Moreteau et al. 1997) but induces high male sterility (Krimbas 1993, see results from “Sterility” experiment). An intermediate temperature of 23.5 °C was included when assessing male sterility to more finely determine broad-sense heritability, as sterility at this temperature varies between populations (Porcelli et al. 2017). Given ongoing climate warming, both elevated temperature treatments are expected to become increasingly common in nature (Calvin et al. 2023), making them ecologically relevant. As the focus of this study was male sterility and female responses, all females used were exposed to 18 °C throughout development (Fig. 1). Once eclosed, virgin focal offspring were collected within 24 h after eclosion under light CO_2_ anaesthesia and kept at 18 °C with the sexes separated: ten females per vial or one male per vial, as group housing decreases male mating probability due to male-male rivalry (Lizé et al. 2014).

### Behavioural observations and trait measurements

Male heat-induced sterility (“Sterility” in Fig. 1) was assessed by pairing one male (18 °C, 23.5 °C or 25 °C) and one female (18 °C) in a vial (Fig. 1) within 30 min of the 12 h light photoperiod to provide a “dawn” stimulus that stimulates peak activity (Shorrocks and Charlesworth 1980). As *D. subobscura* reaches reproductive maturity 6 days post eclosion (Holman et al. 2008), all females used were 6 days old. Males ranged in age from 6 to 16 days, with age included as a covariate to account for age-related increases in fertility (see Statistical Analysis section; Table S1), though sterility remained high across all ages after heat stress with minimal recovery (Fig. S1). Pairs were observed for 3 h (Fisher et al. 2013) in an 18 °C temperature-controlled room under high-intensity light (900–1500 lx; Wallace and Dobzhansky 1946). Non-mating pairs were discarded, while mated females were left for 4 days to lay eggs before being removed. Vials were checked for larvae 8 days after the initial copulation; larvae signified male fertility whereas vials without larvae indicated sterility (Fig. 1). This experiment was conducted twice (“Experimental round” in Statistical Analysis section), with no additional measurements taken.

For all other trait measurements (“Remating” in Fig. 1), 13 isofemale lines were used. One male (18 °C or 25 °C) and one female (18 °C) from the same line were introduced into a vial (“vial 1” in Fig. 1). All experimental individuals were 6 days old (Holman et al. 2008) and mating observations were conducted as above. Non-mating pairs were discarded. If a mating occurred, mating latency (the time from a pair being introduced to copulation beginning) and copulation duration (the length of time from the male copulatory organ entering the female until the male and female disengage) were recorded. After mating, males were discarded and females were left to lay eggs for 4 days (“vial 1”; Fig. 1). After 4 days, each mated female was transferred to a second vial with a new 6 day old control virgin male, as females are most likely to remate after 3 days (Fisher et al. 2013). This new pair was observed for 3 h to determine remating status (Fig. 1). After the observation period, males were discarded and females, regardless of remating status, were left to lay eggs for 4 days (“vial 2” in Fig. 1). After this second oviposition period, females were transferred to a new vial every 7 days and checked every 2 days to monitor survival, but no further offspring were counted. Offspring in vial 1 and 2 were counted at three equally spaced intervals over an 11-day period from when offspring started to eclose (ca. 23 days after mating) to ensure only F1 offspring were counted. Due to the size of the experiment, focal individuals were collected across 6 consecutive days, which were grouped into a single experimental “batch”. The experiment was repeated in 2 rounds, each consisting of 6 batches (see Statistical Analysis section).

Note that the probability of male sterility at either 18 °C or 25 °C was largely consistent between the “sterility” experiment (where only the presence or absence of larvae were noted after mating) and the “remating” experiment (Fig. S2; where offspring number was counted).

### Statistical analysis

All analyses were conducted in R v4.2.2 (R Core Team 2016). Linear models were generated using the ‘lme4’ package v1.1-34, and all figures were created with the ‘ggplot2’ package v3.4.3 (Wickham 2016). Fixed effects and covariates (if included) were evaluated using the *Anova()* function from the ‘car’ package v3.1-2. Post-hoc comparisons of significant main effects were performed using the *emmeans* or *emtrends* function from the ‘emmeans’ package v1.8.8. Random effect significance was evaluated using log-likelihood ratio tests between models including and excluding the variable of interest. Descriptions of all models, model estimates and test statistics are in Tables S1–S5, and sample sizes and mean values are provided in Tables S7–S11.

#### Genetic variation underlying male sterility and female remating

Model descriptions are provided in Tables S1 and S2. Binomial generalised linear mixed models (GLMMs) were used to examine the effect of male development temperature on male sterility and female remating probability. For both models, male development temperature (sterility: 18 °C, 23.5 °C and 25 °C; remating: 18 °C and 25 °C) was included as a fixed effect. Line was included as a random intercept and a random slope that covaried with temperature. The significance of the random slope was tested by comparing models with and without this effect using log-likelihood ratio tests. A significant effect indicates that there is substantial genetic variation underlying a plastic change in response to temperature. When examining sterility, male age was included as a covariate and experimental round (1 and 2) as a random intercept. When examining female remating, batch (1–6) nested within experimental round (1 and 2) was included as a random intercept.

To estimate the proportion of variance explained solely by genetic differences between lines at a given temperature (genetic variance without plasticity), deriving broad-sense heritability (*H*^*2*^), we ran random intercept GLMMs for each temperature treatment (sterility: 18 °C, 23.5 °C and 25 °C; remating: 25 °C). The significance of the intercept for line was tested by comparing models with and without this effect using log-likelihood ratio tests. A significant effect indicates that there is substantial genetic variation in the variable of interest. *H*^*2*^ was calculated using the formula *H*^*2*^ = V_line_/(V_line_ + V_resid_) (Zwoinska et al. 2020) where residual variance is assumed to be π^2/3^ (Nakagawa and Schielzeth 2010). We note that it is not possible to partition the line variance between different genetic components (i.e., additive genetic variance, dominance genetic variance, and epistasis or gene-by-environmental interaction), so this estimate may not accurately reflect the potential for response to selection.

Given significant line-level variation in male fertility at 23.5 °C and female remating probability at 25 °C, we explored the genetic relationship between these traits. Line trait means and standard error were calculated, and a linear model was produced, with each mean weighted by its variance. While this approach does not explicitly test for a genetic correlation, it examines whether genotypes with high sterility probabilities also exhibited high remating probabilities at the line-mean level.

#### Mating probability, mating latency and copulation duration

Model descriptions are provided in Table S3. A binomial GLMM was used to examine the effect of male developmental temperature on the probability of mating, and linear mixed models (LMMs) were used to examine its effect on mating latency and copulation duration for the first mating (Table S3). Male developmental temperature (18 °C and 25 °C) was included as a fixed effect in all models. Random effects included the intercept of batch nested within experimental round and line, allowing the slope to covary with temperature. Mating latency and copulation duration were log- and square-root-transformed to ensure normality.

#### Offspring production

Model descriptions are provided in Table S4. Offspring production was analysed using three linear models using the ‘glmmTMB’ function with a Poisson distribution (Brooks et al. 2017). The first model examined offspring produced in the first vial (after initial mating), the second examined offspring produced in the second vial (after the opportunity to remate), and the third examined total offspring production (vial 1 + vial 2). For all three models, the fixed effect combined the first male’s development temperature and the female’s remating category (male temperature-female mating category: 18 °C monogamous, 18 °C polyandrous, 25 °C monogamous, 25 °C polyandrous), to determine whether female remating after copulating with a thermally stressed male could recover reproductive output. The 25 °C polyandrous male temperature-female mating category was excluded from the first model due to a lack of variance (only one female produced offspring). When examining total offspring production, a zero-inflated model was used to account for vials with zero offspring from the 25 °C polyandrous category in vial 1. For all models, random effects included the intercept of batch nested within experimental round and line, allowing the slope to covary with male temperature-female mating category.

To investigate why females remate after mating with a control male, we ran two follow-up binomial GLMMs (Table S4). First, we tested the effect of male sterility on the probability of female remating. Random effects included the intercept of batch nested within experimental round and line, allowing the slope to covary with the probability of sterility. Next, given a female mated to a fertile control male, we tested how initial reproductive output influenced the probability of remating. For this model, random effects included the intercept of batch nested within experimental round and line.

#### Survival

Model descriptions are provided in Table S5. Survival analysis was performed using the *survfit()* function from the ‘survival’ package v3.5-5 (Therneau 2023) on censored female survival data using the Kaplan-Meir method. Two separate Cox Proportional Hazard regression models tested the fixed effect of male temperature and remating occurrence. Analysing both variables allowed us to differentiate whether the impact of mating with a heat-stressed male on female mortality was driven by polyandry or other factors associated with male heat exposure.

## Results

### Variation in heat-induced male sterility

**Fig. 2:**
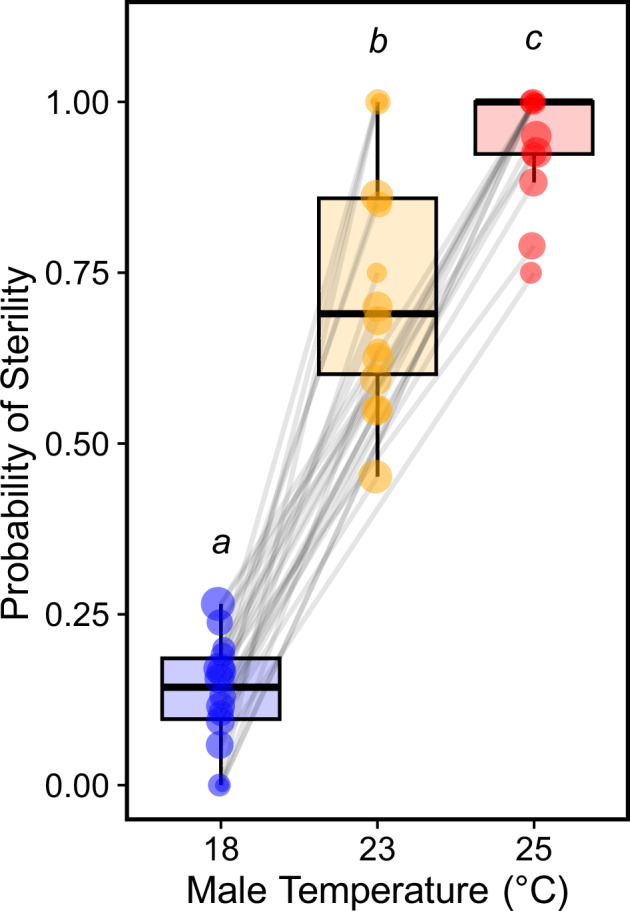
The effect of male development temperature on the probability of sterility. Male sterility increased with rising temperatures. Males that developed at 23.5 °C (orange) and 25 °C (red) were more likely to be sterile compared to control males (blue). Sterility was also greater after mating with a 25 °C male than a 23.5 °C male. Slope variance did not significantly differ between lines but significant genetic variation in male sterility probability was observed at 23.5 °C. Each point is the fraction of matings that were sterile for a given isofemale line, with circle diameter proportional to sample size. Raw values and sample sizes are provided in Table S7.

There was a significant effect of developmental temperature on male sterility (Table S1; ANOVA, *χ2* = 153.35, *df* = 2, *p* < 0.001). Males exposed to 23.5 °C and 25 °C were 1.4 and 1.7 times more likely to be sterile than control males. This corresponds to a 43.3% (Fig. 2; *Estimate* = 3.33 ± 0.39, *z* = 8.52, *p* < 0.001) and 65.9% (Fig. 2; *Estimate* = 5.45 ± 0.46, *z* = 11.80, *p* < 0.001) increase in sterility. Males developed at 25 °C were also 1.2 times more likely to be sterile than 23.5 °C males, representing a ca. 16% increase in sterility (Fig. 2; *Estimate* = 2.11 ± 0.46, *z* = 4.63, *p* < 0.001). Note that not all 18 °C males are fertile, which is consistent with other *Drosophila* species (David et al. 2005), and not all 25 °C males are sterile (Fig. 2).

We assessed genetic variation in fertility plasticity but found no significant slope variance between lines (Fig. 2, Table S1; LRT, *χ2* = 9.81, *p* = 0.133). However, significant line variation in male fertility resilience was observed after exposure to 23.5 °C (intercepts differed; Fig. 2, Table S1; *H*^*2*^ = 0.13; LRT, *χ2* = 10.82, *p* < 0.01), demonstrating broad-sense heritability. As expected, there was limited genetic variation and no significant heritability at either 18 °C (most males were fertile, Fig. 2, Table S1; *H*^*2*^ < 0.01; LRT, *χ2* = 0.01, *p* = 0.941) or 25 °C (most males were sterile, Fig. 2, Table S1; *H*^*2*^ = 0.03; LRT, *χ2* = 0.42, *p* = 0.519).

### Facultative polyandry and its genetic variation

**Fig. 3:**
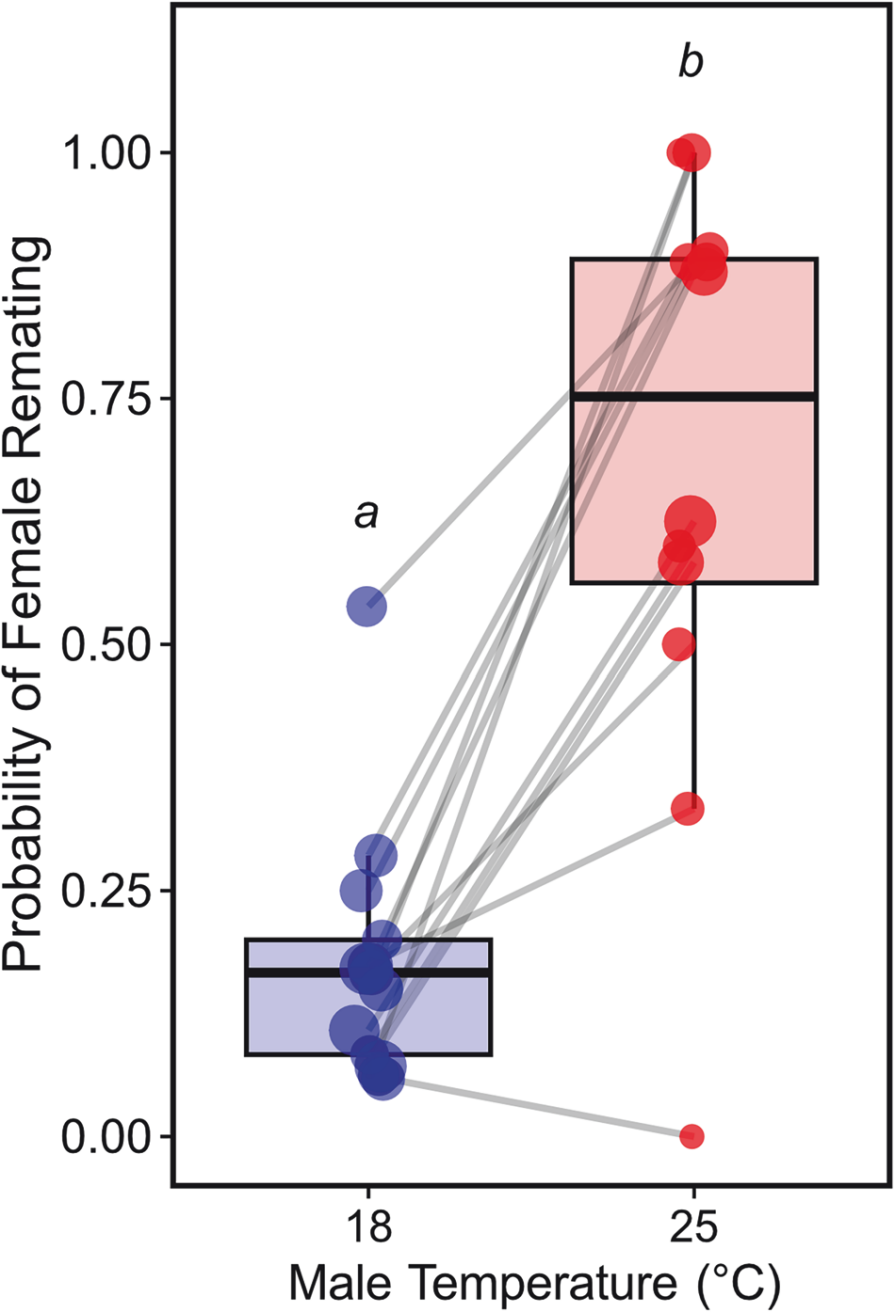
The effect of male development temperature on the probability of female remating when presented with a new control male. The probability of remating was consistently higher after initially mating with heat-stressed males (red) versus initially mating with control males (blue) across all lines (grey slopes). Slope variance did not significantly differ between lines but significant genetic variation in remating probability was observed at 25 °C. Each point represents the fraction of individuals that remated for a given isofemale line, with circle diameter proportional to sample size. The grey line connects points from the same isofemale line. Raw values and sample sizes are provided in Table S8.

Facultative polyandry increased when females initially mated a heat-stressed male compared to a control male (18 °C monogamous: *n* = 230, 18 °C polyandrous: *n* = 46, 25 °C monogamous: *n* = 37, 25 °C polyandrous: *n* = 106, Table S2; ANOVA, *χ2* = 3.17, *df* = 1, *p* < 0.001), with females 1.5 times more likely to remate – a 49.25% increase in remating probability compared to controls (Fig. 3; *Estimate* = 3.17 ± 0.41, *z* = 7.82, *p* < 0.001). This increase in remating probability was consistent across isofemale lines (i.e., no slope variance between lines; Fig. 3, Table S2; LRT, *χ2* = 2.24, *p* = 0.525), suggesting little genetic variation in this plastic response. Although there was a lack of genetic variation in plasticity, some lines were more polyandrous than others (intercepts differed; Fig. 3, Table S2; *H*^*2*^ = 0.21; LRT, *χ2* = 5.35, *p* < 0.05), providing evidence for between line variation in facultative polyandry. Together, these results suggest that, while all lines plastically respond to mating with heat-stressed males by elevating remating compared to when mating with control males, their tendency to remate varies genetically.

Finally, we explored the relationship between male fertility tolerance and facultative polyandry at the line mean level. We used male data at 23.5 °C given we saw more genetic variation at this temperature. There was no significant correlation when either all lines were included in the analysis (Fig. S3; *Estimate* = −0.60 ± 0.33, *t* = −1.84, *p* = 0.097) or when only lines displaying variance for both traits were included (*Estimate* = 0.10 ± 0.40, *t* = 0.26, *p* = 0.806), indicating no genetic association between male sterility and female remating.

### Male heat stress effects on mating behaviour

**Fig. 4:**
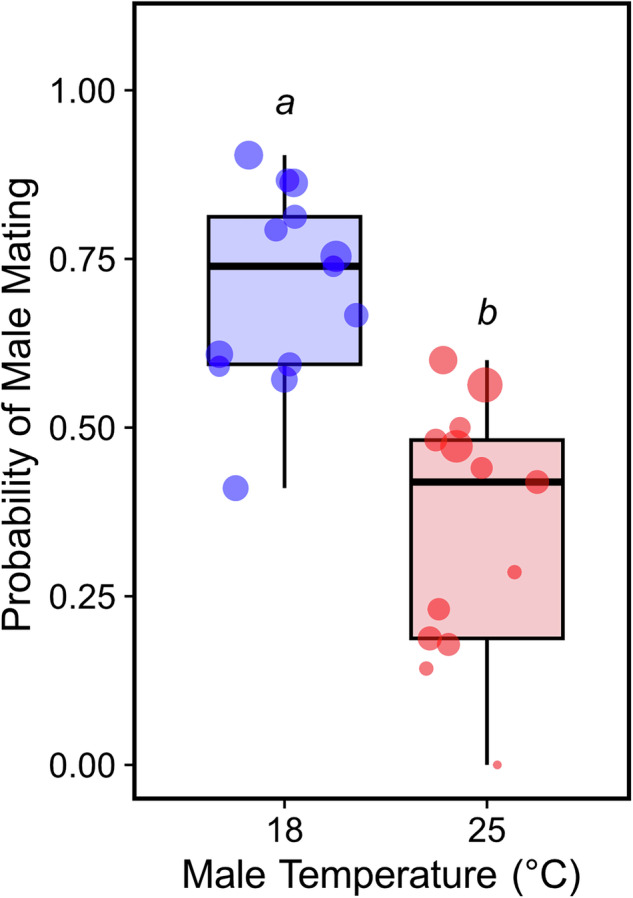
The effect of male development temperature on the probability of an initial mating. Heat-stressed males (red) were less likely to mate than control males (blue). Each point is the fraction of individuals that mated for a given isofemale line, with circle diameter proportional to sample size. Raw values and sample sizes are provided in Table S8. Note that values for 18 °C males are are typical of this species (Fisher et al. 2013).

Male heat stress significantly impacted male mating probability (Table S3; ANOVA, *χ2* = −1.85, *df* = 1, *p* < 0.001); heat-stressed males were 1.2 times less likely to mate compared to control males (Fig. 4; *Estimate* = −1.85 ± 0.23, *z* = −8.11, *p* < 0.001), which is a 19.5% decrease in mating probability. When heat-stressed males did mate, both mating latency (Table S3; *Estimate* = 1.03 ± 0.14, *z* = 7.53, *p* < 0.001) and copulation duration (Table S3; *Estimate* = 0.60 ± 0.13, *z* = 4.72, *p* < 0.001) were significantly longer compared to control males.

### Fitness effects of monandry and facultative polyandry

**Fig. 5:**
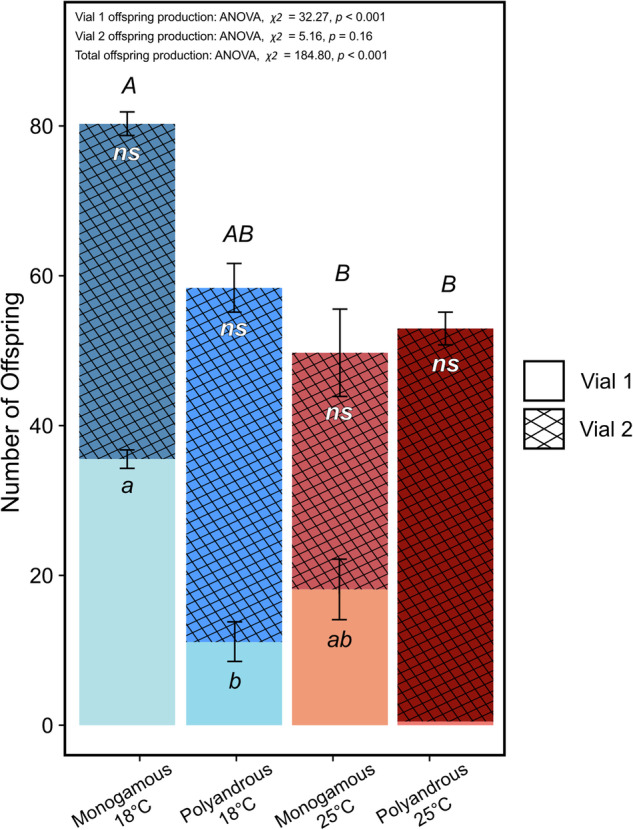
The effect of male development temperature on the number of offspring produced after two mating opportunities. Each male temperature-female mating category (left to right: monogamous female mated to an 18 °C male, polyandrous female mated to an 18 °C male, monogamous female mated to a 25 °C male, polyandrous female mated to a 25 °C male) is represented by a stacked bar which includes the offspring produced in vial 1 (initial mating; plain filled bar) and vial 2 (after the opportunity to remate with a new 18 °C male; hatched bar). Almost no offspring were produced in vial 1 by females mated to 25 °C males that went onto remate (polyandrous 25 °C). Monogamous females mated to 18 °C males produced significantly more offspring in vial 1 than polyandrous females initially mated to 18 °C males. The number of offspring produced by females in vial 2 (after the opportunity to remate) did not significantly differ between male temperature-female mating groups (as indicated by “*ns*” within the hatched bar). Overall, monogamous females mated to 18 °C males produced significantly more offspring than remating females. Significant differences between categories for vial 1 and 2 are shown within the bar (lower case letters), and total reproductive output (vial 1 + vial 2) is shown above the bar (upper case letters). Raw values and sample sizes are provided in Table S9.

Our experimental design allows for four male temperature-female mating categories (18 °C monogamous, 18 °C polyandrous, 25 °C monogamous, 25 °C polyandrous). We expected females mated to 18 °C males would fall in the category of 18 °C monogamous, whereas females mated to 25 °C males would fall into two categories: 25 °C polyandrous (because some females facultatively remate) and 25 °C monogamous (because not all females remate and not all males are sterile (Fig. 3, Fig. S2). Surprisingly, there were some females initially mated to 18 °C males that remated (18 °C polyandrous category; Fig. 5). The probability of facultative polyandry increased here because control males were either sterile (Fig. S2, Table S4, S10; *Estimate* = 3.38 ± 0.50, *z* = 6.73, *p* < 0.001) or produced sub-fertile ejaculates, given the production of few offspring (Table S4, S11; *t* = 0.04 ± 0.01, *z* = 4.93, *p* < 0.001).

Offspring production after the initial mating but prior to a remating opportunity (vial 1) differed between male temperature-female mating categories (Fig. 5, Table S4; ANOVA, *χ2* = 32.27, *df* = 2, *p* < 0.001). Monogamous females produced more offspring than polyandrous females when initially mated to 18 °C males (Fig. 5; Table S6). Monogamous females mated to 25 °C males produced an intermediate number of offspring compared to these two categories, although sample size is low so interpreting this pattern requires caution (Fig. 5, Table S6). However, in vial 2, after the opportunity to remate, facultatively polyandrous females matched offspring production of monogamous females (Fig. 5, Table S6). Overall, total progeny production was greater for monogamous females receiving an adequate ejaculate, driven by their continuous production of offspring across the first and second vial (Fig. 5, Table S6; ANOVA, *χ2* = 184.8, *df* = 3, *p* < 0.001).

**Fig. 6:**
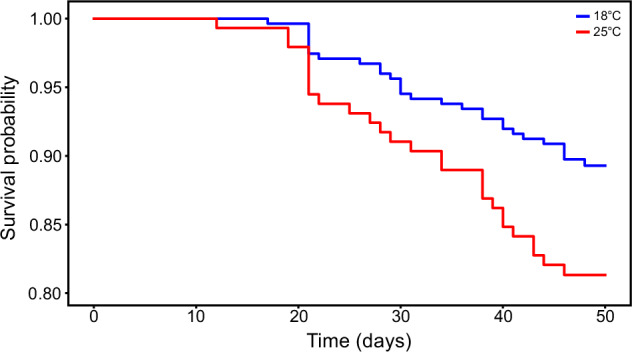
The effect of male development temperature on female mortality. Females mated to heat-stressed males (red) had a significantly reduced lifespan than females mated to control males (blue). Survival probability describes the fraction of individuals that were alive for a given temperature treatment across all lines. Time describes the number of days post initial mating opportunity.

In addition to lower total offspring number, mating with a heat-stressed male reduced female longevity compared to a mating with a control male (Fig. 6, Table S5; Hazard Cox: *χ2* = 5.31, *df* = 1, *p* < 0.05). This effect was not driven by higher instances of remating as there was no significant difference in survival between monogamous and polyandrous females (Hazard Cox: *χ2* = 0.73, *df* = 1, *p* = 0.394).

## Discussion

Developmental heat stress caused severe male sterility, aligning with findings in other *D. subobscura* populations (Porcelli et al. 2017; Santos et al. 2023; Grandela et al. 2024) and other *Drosophila* species (e.g., David et al. 2005; Green et al. 2019; Zwoinska et al. 2020; Rodrigues et al. 2022). Sterility probability increased as temperature increased across all lines, showing limited genetic variation in sterility plasticity. At 23.5 °C, however, phenotypic variation between lines was sufficient to generate broad-sense heritability, providing some evidence of evolutionary potential in male fertility resilience to moderate temperature stress. Facultative polyandry also increased across lines following a mating with a heat-sterilised male compared to a control male, aligning with other work using a different genetic background (Fisher et al. 2013). Although this behavioural plasticity showed no genetic variation, lines significantly varied in heritable polyandry (i.e., some lines are more polyandrous than others). Therefore, climate-driven increases in heat-induced male sterility may favour polyandry-associated genes, potentially increasing polyandry in this system. While heritable variation in both female remating propensity and male fertility resilience to heat stress may imply that high female polyandry in certain lines is driven by high male fertility sensitivity in that line, we found no significant correlation between traits at the line-mean level, suggesting independent evolution of sex-specific traits. This species has clinally varying chromosomal inversion polymorphisms (Krimbas 1993), some of which influence heat adaptation and response to climate-driven environmental changes (Balanyá et al. 2006; Rezende et al. 2010; Rodríguez-Trelles and Tarrío 2024). Whether these inversions contain genes that are important for either of these two traits is unknown. Investigating the genetic relationship between, and genes underlying, both traits would significantly advance our understanding of how male thermal physiology and mating systems, shaped by female remating behaviour for fertility assurance, can shift in response to rising temperatures.

Remating increased offspring production when an initial copulation involved a sterile or sub-fertile male. These results align with those in polyandrous species, showing remating can serve as fertility assurance (e.g., Price et al. 2008; Sutter et al. 2019; Vasudeva et al. 2021). Thus, even in typically monandrous species, direct benefits of remating following a copulation with a sterile or sub-fertile male may drive selection for polyandry. However, while polyandry for fertility assurance provides an obvious fitness benefit, we found that facultatively polyandrous females could not completely mitigate the cost of their first mate’s sterility, leading to lower total offspring production. One caveat is that, as females were only allowed to lay eggs for 3 days after remating despite their capacity to lay eggs over a longer period, remated females may fully recover fertility if we had measured progeny production for longer. Females mated to heat-stressed males also had shorter lifespans, which may be the result of intensified sexual conflict through mechanisms such as male harassment or changes in ejaculate toxicity (García-Roa et al. 2020). However, this lifespan reduction is unlikely to be ecologically relevant for wild *D. subobscura*, which are estimated to live for 26 days (Junge-Berberović, 1996), as the divergence in survival observed here only became apparent after approximately 22 days.

The mechanisms triggering female remating after an initial mating with a heat-stressed male are unknown. We suggest four, non-mutually exclusive hypotheses to explain the observed increase in remating probability. First, heat-stressed males copulate longer, a behaviour linked to deteriorating male condition and exhaustion in this species (Lizé et al. 2014), which may serve as a female remating cue. Second, *D. subobscura* males donate nutritious nuptial gifts that improve female reproductive success (Steele 1986; Immonen et al. 2009; Pembury Smith et al. 2025). As heat stress reduces male gift giving (Grandela et al. 2024; Pembury Smith et al. 2025), females may remate following inadequate male-donated nutrition from their first mating. Third, polyandry could be triggered by a lack of sperm in storage if females can detect sperm load, as both sterile and sub-fertile matings increased remating propensity. Fourth, heat stress may impact the transfer of seminal fluid proteins (SFPs) which are known to influence female remating in *D. melanogaster* (Canal Domenech and Fricke 2022). One intriguing SFP is *Sex Peptide* (SP) that attaches to sperm after transfer and its gradual cleavage suppresses remating (Liu and Kubli 2003; Peng et al. 2005). Two copies of SP are present in the *D. suboscura* genome. Both are transcribed, exhibit signatures of positive selection, and the cleavage site is conserved with *D. melanogaster*, suggesting a similar function in the two species (Cirera and Aguadé, 1998). If sterile males transfer no sperm, then SP has nothing to bind to, preventing long term suppression of female remating. We are currently testing these hypotheses.

A switch to polyandry would increase sexual conflict (Holman and Kokko 2013; Perry and Rowe 2018). As an alternative to polyandry, females could increase mate discrimination. Lowered mating probability and longer latencies are signatures of female mate rejection (Taylor et al. 2008), and we found male heat stress negatively impacted both traits. Moreover, exposure to high developmental temperatures impairs male ability to produce nuptial gifts (Grandela et al 2024; Pembury Smith et al. 2025), which could signal poor male quality to females. Generally, pre-copulatory choice in monogamous species may be strong as post-copulatory mechanisms of cryptic female choice and sperm competition – which may be costly – are presumed to be rare (Hosken et al. 2009). Female *D. subobscura* display pre-copulatory discrimination against starved (Steele 1986; Immonen et al. 2009), irradiated (Savic Veselinovic et al. 2017), and old males (Verspoor et al. 2015), although the mechanisms underlying this discrimination are unknown. Avoiding a copulation with a heat-stressed male would provide a direct benefit to females as it reduces the risk of fertility loss. However, increased female mate discrimination may be costly (e.g. increased energy costs, predation risk; Dougherty 2021) and fertile males may become sperm depleted (Wedell et al. 2002; Linklater et al. 2007; Hasson and Stone 2009), undermining fitness benefits of female mate discrimination. Future research examining the extent of evolutionary change in either male fertility tolerance, female pre-copulatory discrimination and/or prevalence of polyandry under warming temperatures would provide insight on the dynamics of fertility assurance, mate choice and sexual conflict, improving understanding of how mating systems may respond to climate change.

Selection for increased polyandry or mate discrimination may be weakened if male fertility becomes more robust to thermal stress. As male fertility responses to heat stress exhibited broad-sense heritability, selection may favour thermally resilient males, reducing sterility and decreasing selection for polyandry. However, prior experimental evolution studies in *D. subobscura* found no clear evidence that traits indirectly linked to male fertility improved under warming conditions (Santos et al. 2023). This aligns with work in other *Drosophila* species that have failed to demonstrate evolutionary potential in upper male fertility limits in response to elevated temperatures (van Heerwaarden and Sgrò 2021). Given current evidence, substantial evolutionary improvement in male thermal fertility tolerance to climate warming appears unlikely. However, these studies imposed strong selection regimes (Santos et al. 2023; van Heerwaarden and Sgrò 2021). We observed genetic variation for male fertility resilience only under exposure to moderate temperatures, so perhaps opportunity for genetic response was limited in the approaches used. Moreover, we found genetic variation in polyandry under higher temperatures, and not all 25 °C treated males were sterile; thus females may recover reproductive output via remating, so selection on polyandry may still play a critical role in buffering fertility loss. Future experimental evolution studies using less severe thermal regimes that directly examine the relationship between male fertility tolerance and female remating behaviour would be useful.

This study offers valuable insights into heat stress effects on male fertility and female remating, however, several limitations should be considered. First, only males were heat stressed. In the wild, both sexes likely experience similar thermal environments during development, so assessing how female thermal stress impacts female remating behaviour should be explored for a more complete understanding of temperature impacts on mating dynamics. Thermally stressed females could be less likely to remate if their energy reserves are depleted, or more likely to remate in order to receive additional nuptial gifts that may offset thermally-induced somatic and reproductive costs (Pembury Smith et al. 2025). Second, while within-line matings allow standardising genetic background to detect differences between lines, this approach limits the ability to identify sex-specific genetic contributions to the observed variation, and may conflate genetic variation with inbreeding effects. Future work incorporating between-line crosses will help disentangle these impacts.

## Conclusion

Our goal was to assess genetic variation in male thermal fertility tolerance and facultative polyandry. Since *D. subobscura* females have been shown to remate when they receive a sub-fertile ejaculate (Fisher et al. 2013), we needed to generate substantial male sterility to test whether facultative polyandry shows heritable variation. We achieved this using heat stress, an ecologically relevant variable given climate change. Heat stress reliably induced male sterility, revealing genetic variation in male fertility tolerance, albeit only at moderate temperatures. In response to receiving a heat-induced sterile or sub-fertile ejaculate, females facultatively remated, a behavioural shift that also exhibited line-specific genetic variation, and allowed females to mitigate some of the fitness costs associated with male infertility. Therefore, as global temperatures rise and heat-induced male sterility increases, selection for polyandry may be promoted in this typically monandrous system. Further exploring this possibility, along with potential evolutionary shifts in male fertility tolerance, is essential to understanding how reproductive traits and mating systems evolve under climate change, with important implications for population dynamics and viability (Holman and Kokko 2013).

## Supplementary information

Supplementary Material

## Data Availability

Data and code used in this study are available on Dryad 10.5061/dryad.1rn8pk173.
