# Supplementary material for: Facultative polyandry under heat stress and the evolutionary potential for climate-driven shifts in mating systems
Source: Heredity (Edinb). 2025 Sep 18;134(10-11):596–604. doi: 10.1038/s41437-025-00795-5 (PMC12595111; doi:10.1038/s41437-025-00795-5)
Supplement: Supplementary file 1 — Supplementary Material [file 41437_2025_795_MOESM1_ESM.pdf]

Supplementary Material

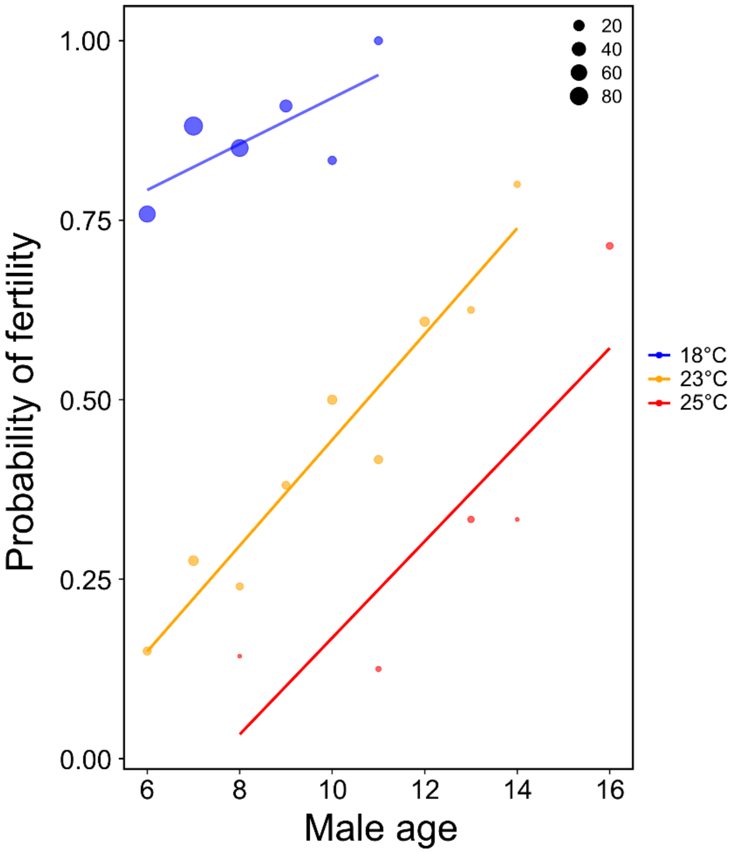

Figure S1. The proportion of fertile males age 6 – 16 after exposure to each temperature treatment. The proportion of fertile individuals increased with age. However, this relationship did not significantly differ between temperature treatment groups, and high male sterility was observed across all age groups after heat-exposure. Colour represents male development temperature. Each point represents the fraction of individuals that were fertile for a given age group, with circle diameter proportional to sample size.

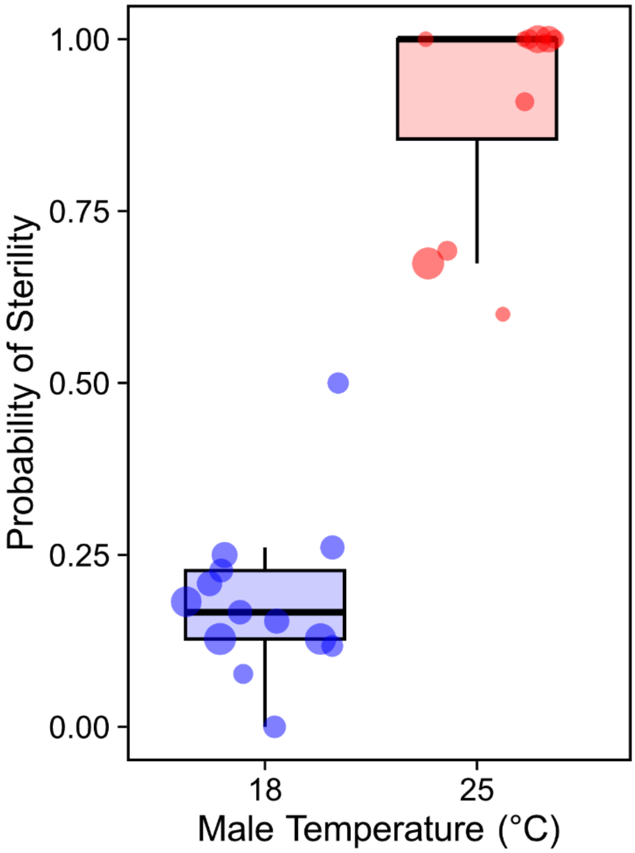

Figure S2. The effect of male developmental temperature on the probability of sterility from "remating" experiment. Male sterility was substantially higher when males were heat-stressed (red) compared to control males (blue). This result, taken from the "remating" experiment was consistent with the results from the "sterility" experiment. Each point is the fraction of individuals that were sterile forgiven isofemale line, with circle diameter proportional to sample size. Raw values and sample sizes are provided in Table S8.

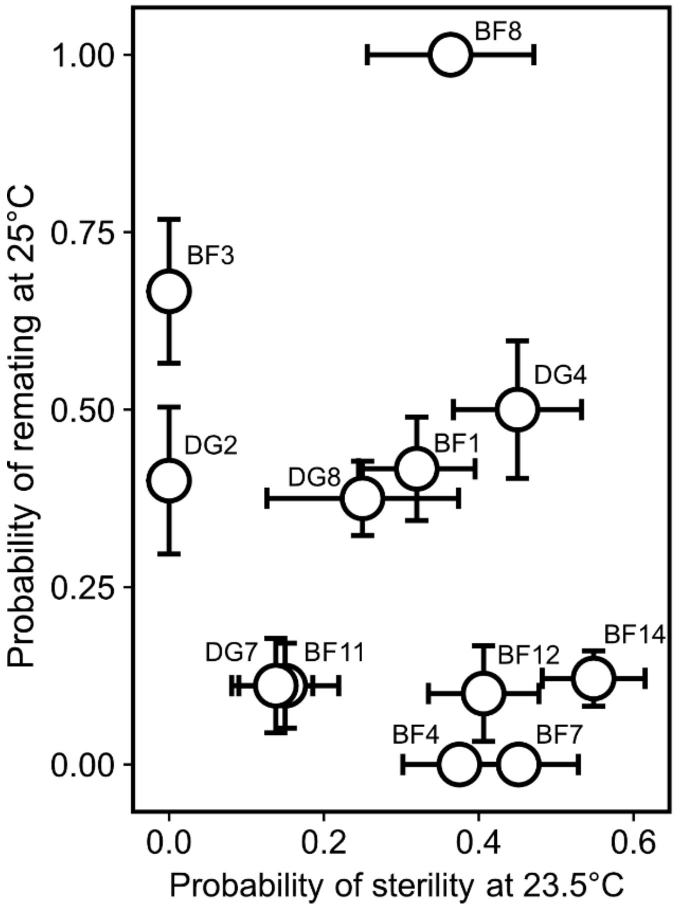

Figure S3. The relationship between the probability of sterility and the probability of remating at the line level. As the probability of sterility at 23.5°C and the probability of remating at 25°C displayed genetic variation, we examined the relationship between these two probabilities at the isofemale line level. Lines that displayed stronger fertility sensitivity to temperature did not correlate with lines that had a higher probability of remating. Each point represents the mean ± standard error for each line.

Table S1. Description of the statistical models used to examine male sterility. First, we assessed the effect of development temperature (18°C, 23.5°C or 25°C) on the probability of male sterility. Random effects included (Temp|Line), (1|Line) and (1|Experiment). The variance and standard deviation of the intercept was calculated for each random effect. For the random effect (Temp|Line), we also calculated the variance and standard deviation of the slope (comparing a change in the probability of sterility from 18°C to 23.5°C or 25°C), and its overall significance was evaluated using a log-likelihood ratio test to assess genetic variance in sterility plasticity. Separate random intercept models for each temperature were then used to quantify variability at each temperature and to derive broad-sense heritability. Variance of line was derived from the full model and the significance of the intercept for line was evaluated using a log-likelihood ratio test to assess genetic variance in sterility. "Temp" refers to male development temperature (18°C, 23.5°C or 25°C). "Line" is the isofemale line. "Experiment" describes the random effect of experiment (1 and 2). In "Fixed and Random Effects", fixed effects are upright, random effects are in italics. VAR/SD is the variance and standard deviation of the random effect (intercept or slope). The *p* value for a fixed effect, covariate or random effect indicates its significance from an ANOVA or log-likelihood ratio test. Significant effects are in bold.

Var of interest		Model	Family	Fixed and Random Effect		X^2	*VAR/SD*	*p*
**The probability of sterility**		**Temp + Male Age + (Temp| Line) + (1|Line) + (1|Experiment)**	**Binomial**	**Temp**		**153.35**		**<0.001**
				**Male Age**		**55.19**		**< 0.001**
				Temp | Line	Intercept		0.03/0.18	
					Temp 23.5°C		0.76/0.87	
					Temp 25°C		0.02/0.15	
Genetic variance in sterility plasticity						9.81		0.133
				Line			<0.001/<0.001	
				Experiment			<0.001/<0.001	
Genetic variance in sterility	*18°C*	(1| Line) + (1|Experiment)	Binomial	*Line*		0.01	0.01/0.08	0.941
				Experiment			<0.001/<0.001	
	23.5°C	**(1| Line) + (1|Experiment)**		***Line***		**10.82**	**0.61/0.78**	**<0.01**
				Experiment			0.65/0.81	
	25°C	(1| Line) + (1|Experiment)		*Line*		0.42	0.36/0.60	0.519
				Experiment			7.60/2.76	

Table S2. Description of the statistical models used to examine female remating propensity. First, we assessed the effect of the development temperature of the first male (18°C or 25°C) on the probability of female remating. Random effects included (Temp|Line), (1|Line) and (1|Experiment). The variance and standard deviation of the intercept was calculated for each random effect. For the random effect (Temp|Line), we also calculated the variance and standard deviation of the slope (comparing a change in the probability of remating from mating with a male developed at 18°C to a male developed at 25°C), and its overall significance was evaluated using a log-likelihood ratio test to assess genetic variance in polyandry plasticity. A separate random intercept model then used to quantify genetic variability at 25°C and to derive broad-sense heritability. Variance of line was derived from the full model and the significance of the intercept for line was evaluated using a log-likelihood ratio test to assess genetic variance in polyandry. "Temp" refers to male development temperature (18°C or 25°C). "Line" is the isofemale line. "Round:Batch" describes the experimental batches that occurred within two experimental rounds. In "Fixed and Random Effects", fixed effects are upright, random effects are in italics. VAR/SD is the variance and standard deviation of the random effect (intercept or slope). The *p* value for a fixed effect, covariate or random effect indicates its significance from an ANOVA or log-likelihood ratio test. Significant effects are in bold.

Var of interest		Model	Family	Fixed and Random Effect		X^2	*VAR/SD*	*p*
**The probability of female remating**		**Temp + (Temp| Line) + (1| Line) + (1|Round) + (1|Round:Batch)**	**Binomial**	**Temp**		**3.17**		**< 0.001**
				Temp | Line	Intercept		0.03/0.17	
					Temp 25°C		0.46/0.69	
Genetic variation in polyandry plasticity						2.24		0.525
				Line			<0.001/<0.001	
				Round:Batch			0.71/0.84	
				Round			<0.001/<0.001	
Genetic variation in polyandry	***25°C***	**(1| Line) + (1|Round) + (1|Round:Batch)**	**Binomial**	***Line***		**5.35**	**1.06/1.03**	**<0.05**
				Round:Batch			0.74/0.86	
				Round			<0.001/<0.001	

Table S3. Description of the statistical models used to examine additional behavioral traits during the initial mating: the probability of mating, mating latency and duration. "Temp" is the male development temperature (18°C or 25°C). "Line" is the isofemale line. In "Fixed and Random Effects", fixed effects are upright, random effects are in italics. "Round:Batch" describes the experimental batches that occurred within two experimental rounds. VAR/SD is the variance and standard deviation of the random effect (intercept or slope). The *p* value describes the significance of the fixed effect from an ANOVA analysis. Significant effects are in bold.

Var of interest	Model	Family	Fixed and Random Effect		*Estimate* ± SE	*VAR/SD*	*p*
**Mating**	**Temp + (Temp| Line) + (1|Round) + (1|Round:Batch)**	**Binomial**	**Temp**		**-1.85 ± 0.23**		**< 0.001**
			Temp | Line	Intercept		0.48/0.69	
				Temp 25°C		0.17/0.42	
			Round:Batch			0.003/0.06	
			Round			0.52/0.719	
**Mating Latency**	**Temp + (Temp| Line) + (1|Round) + (1|Round:Batch)**	**Gaussian**	**Temp**		**-1.03 ± 0.14**		**< 0.001**
			Temp | Line	Intercept		0.08/0.28	
				Temp 25°C		0.03/0.17	
			Round:Batch			0.06/0.24	
			Round			0.10/0.31	
**Copulation Duration**	**Temp + (Temp| Line) + (1|Round) + (1|Round:Batch)**	**Gaussian**	**Temp**		**0.60 ± 0.13**		**< 0.001**
			Temp | Line	Intercept		0.08/0.28	
				Temp 25°C		0.09/0.30	
			Round:Batch			0.02/0.13	
			Round			0.05/0.22	

Table S4. Description of the statistical models used to examine fitness traits. "Remating_Temp" is the male temperature-female mating category (18°C monogamous, 18°C polyandrous, 25°C monogamous, 25°C polyandrous). Offspring produced in vial 1 is the number of offspring produced after the initial mating. No offspring were produced by females mated to 25°C males that went on to remate so this group was not included in this analysis. Offspring produced in vial 2 is the number of offspring produced after giving a female the opportunity to remate. The total number of offspring produced is the number of offspring from vial 1 + vial 2. Following this analysis, we examined the effect of sterility on the probability of remating for females only mated to 18°C males. "Sterility" is whether an individual was sterile (yes, no). We also examined the effect of initial offspring production (those produced in vial one after the initial mating) on the probability of remating for females mated to fertile 18°C males. "Vial 1 offspring" is the total number of offspring produced after the initial mating. "Line" is the isofemale line. In "Fixed and Random Effects", fixed effects are upright, random effects are in italics. "Round:Batch" describes the experimental batches that occurred within two experimental rounds. VAR/SD is the variance and standard deviation of the random effect (intercept or slope). The *p* value describes the significance of the fixed effect from an ANOVA analysis. Significant effects are in bold.

Var of interest	Model	Family	Fixed and Random Effect		X^2/*Estimate* ± SE	*VAR/SD*	*p*
**Offspring produced in vial 1**	**Remating_Temp + (Remating_Temp | Line) + (1|Round) + (1|Round:Batch)**	**Poisson**	**Remating_Temp**		**32.27**		**<0.001**
			Remating_Temp | Line	*Intercept*		0.04/0.19	
				18_P		3.09/1.76	
				25_P		35.03/6.00	
				Round:Batch		<0.001/<0.001	
				Round		0.02/0.15	
**Offspring produced in vial 2**	**Remating_Temp + (Remating_Temp | Line) + (1|Round) + (1|Round:Batch)**	**Poisson**	Remating_Temp		**5.16**		**0.16**
			Remating_Temp|Line	*Intercept*		0.06/0.25	
				18_P		0.10/0.31	
				25_M		21.47/4.6	
				25_M		0.11/0.34	
				Round:Batch		0.01/0.08	
				Round		0.01/0.11	

Table S4. (continued)

Var of interest	Model	Family	Fixed and Random Effect		X^2/*Estimate* ± SE	*VAR/SD*	*p*
**Total number of offspring produced**	**Remating_Temp + (Remating_Temp | Line) + (1|Round) + (1|Round:Batch)**	**Zero-inflated poisson**	**Remating_Temp**		**184.80**		**<0.001**
			Remating_Temp**|Line**	*Intercept*		0.04/0.20	
				18_P		0.04/0.12	
				25_M		0.58/0.76	
				25_M		0.04/0.20	
			Round:Batch			0.004/0.06	
			Round			0.01/0.07	
**The probability of remating (only when mating with a control male)**	**Sterility + (Sterility | Line) + (1|Round) + (1|Round:Batch)**	**Binomial**	**Sterility**		**3.55 ± 0.53**		**<0.001**
			Sterility | Line	*Intercept*		0.17/0.40	
				Sterility Y		0.01/0.01	
			Round:Batch			1.49/1.22	
			Round			0.02/0.04	
**The probability of remating (only when mating with a fertile control male)**	**Vial 1 offspring + (1 | Line) + (1|Round) + (1|Round:Batch)**	**Binomial**	**Vial 1 offspring**		**-0.04 ± 0.001**		**<0.001**
			Line			0.42/0.65	
			Round:Batch			0.48/0.69	
			Round			<0.001/<0.001	

Table S5. Description of the statistical models used to examine survival. Survival was evaluated by using the *Surv()* function. The model estimates the Hazard Ratio which quantifies the effect of male temperature treatment on survival time. The response variable was Surv(Day, Event) where Day represents the time variable (e.g., age of individual), and Event is a binary indicator variable (1 if death occurred, 0 if censored). Data was censored if an individual was still alive at the end of the experiment. "Temp" is the male development temperature (18°C or 25°C). "Remating" is whether the female remated or not (yes or no). *Coef* is the estimated regression coefficient for the predictor, *exp(coef)* is the exponentiated coefficient which represents how much the risk of death changes per change in fixed effect. *Se(coef)* describes the uncertainty around the estimated coefficient. The *z* value is the test statistic and the *p* value describes the significance of the fixed effect. Significant effects are in bold.

Var of interest	Model	*coef*	*Exp(coef)*	*se(coef)*	*z*	*p*
**Survival**	**Temp**	**0.089**	1.093	**0.038**	**2.33**	**<0.05**
Survival	Remating	0.256	1.292	0.299	0.856	0.392

Table S6. Posthoc test statistics for offspring analysis. We compared the number of offspring the female produced between male temperature-female remating categories (18°C monogamous, 18°C polyandrous, 25°C monogamous, 25°C polyandrous). Vial 1 describes the number of offspring produced in the first vial (after the initial mating). For this analysis, no offspring were produced in females mated to 25°C that went on to remate so this category could not be compared. Vial 2 describes the number of offspring produced in the second vial (after the opportunity to remate). Total describes the total number of offspring the female produced (vial 1 + vial 2). "Mono" = monogamous, "P" = polyandrous. For models for these analyses see Table S4. Significant effects are in bold.

Offspring number	Temperature remating category		Estimate	SE	*z*	*p*
**Vial 1**	**18°C Mono**	**18°C P**	**2.68**	**0.56**	**4.78**	**<0.001**
	18°C P	25°C Mono	8.54	3.73	2.23	0.06
	18°C P	25°C Mono	5.86	3.87	1.52	0.28
Vial 2	18°C Mono	18°C P	-0.08	0.09	-0.85	0.82
	18°C Mono	25°C Mono	5.01	2.38	2.11	0.15
	18°C Mono	25°C P	-0.09	0.10	-0.83	0.84
	18°C P	25°C Mono	5.09	2.38	2.14	0.14
	18°C P	25°C P	-0.01	0.09	0.08	0.99
	25°C Mono	25°C P	-5.09	2.40	2.13	0.14
**Total**	**18°C Mono**	**18°C P**	**0.47**	**0.06**	**8.10**	**<0.001**
	18°C Mono	25°C Mono	0.52	0.31	1.67	0.34
	**18°C Mono**	**25°C P**	**0.64**	**0.07**	**9.39**	**<0.001**
	18°C P	25°C Mono	0.05	0.34	0.16	0.99
	18°C P	25°C P	0.17	0.08	2.12	0.15
	25°C Mono	25°C P	0.12	0.33	0.35	0.99

Table S7. The individual line sample sizes for each male temperature treatment when analysing the probability of sterility following development exposure to 18°C, 23.5°C or 25°C.

Temp	Mean ± SE	BF1	BF11	BF12	BF14	BF3	BF4	BF7	BF8	DG1	DG2	DG3	DG4	DG7	DG8
18°C	0.16 ± 0.02	32	21	49	32	19	21	23	4	15	12	18	26	41	17
23.5°C	0.66 ± 0.02	25	20	32	31	2	24	31	11	3	6	10	20	29	8
25°C	0.92 ± 0.02	4	15	14	20	5	13	27	6	4	6	1	17	19	12

Table S8. The individual line sample sizes for each male temperature treatment. Row header describes the response variable for the test conducted. The frequency in the remating analysis is less than those in the mating latency/copulation duration analysis when not enough control males were available.

Line	Temp	Mating (N/Y)	Mating Latency	Copulation Duration	Remating (N/Y)	Sterility (18 vs 25)
BF1	18°C	65 (16/49)	47	47	28 (26/2)	47
	25°C	50 (20/30)	30	30	12 (5/7)	30
BF11	18°C	33 (11/22)	22	22	20 (15/5)	22
	25°C	31 (18/13)	11	10	9 (1/8)	13
BF12	18°C	29 (6/23)	23	23	21 (15/6)	23
	25°C	22 (11/11)	11	11	10 (1/9)	11
BF14	18°C	52 (5/47)	46	46	41 (34/7)	47
	25°C	72 (38/34)	33	32	33 (4/29)	33
BF3	18°C	46 (18/28)	27	27	23 (19/4)	28
	25°C	26 (20/6)	6	6	6 (4/2)	6
BF4	18°C	42 (18/24)	23	23	17 (16/1)	24
	25°C	7 (5/2)	2	2	2 (0/2)	2
BF7	18°C	32 (6/26)	22	22	20 (17/3)	26
	25°C	27 (14/13)	12	12	10 (0/10)	12
BF8	18°C	32 (13/19)	18	18	16 (15/1)	19
	25°C	7 (6/1)	1	1	1 (3/1)	1
DG2	18°C	22 (9/13)	13	13	12 (11/1)	13
	25°C	28 (23/5)	5	5	5 (2/3)	5
DG3	18°C	39 (23/16)	16	16	10 (8/2)	16
	25°C	3 (3/0)	NA	NA	NA	NA
DG4	18°C	30 (4/26)	25	25	18 (15/3)	24
	25°C	32 (26/6)	6	6	6 (3/3)	6
DG7	18°C	23 (6/17)	15	15	13 (6/7)	17
	25°C	25 (14/11	10	10	9 (1/8)	9
DG8	18°C	51 (7/44)	43	43	37 (33/4)	44
	25°C	87 (38/49)	47	47	40 (15/25)	46
**Overall**		**913**	**514**	**512**	**419**	**524**
Overall Mean	18°C	0.71 ± 0.02	20.20 ± 1.57	15.70 ± 0.81	0.17 ± 0.02	0.18 ± 0.02
	25°C	0.43 ± 0.02	35.50 ± 2.63	52.20 ± 9.23	0.74 ± 0.03	0.87 ± 0.02

Table S9. The individual line sample sizes for each male temperature-female mating category when analysing the number of offspring produced in vial 1, vial 2 and the total (vial 1 + vial 2). "Mono" = monogamous, "P" = polyandrous.

Vial	Temp	Mean ± SE	Total	BF1	BF11	BF12	BF14	BF3	BF4	BF7	BF8	DG2	DG3	DG4	DG7	DG8
Vial 1	18°C Mono	36.10 ± 1.26	223	25	14	14	33	17	16	17	14	11	8	14	6	33
	18°C P	9.96 ± 2.41	45	2	5	5	7	4	1	3	1	1	2	3	7	4
	25°C Mono	19.70 ± 4.33	34	4	1	1	3	4	NA	NA	1	2	NA	3	1	14
Vial 2	18°C Mono	44.70 ± 1.62	223	25	15	14	33	17	16	17	14	11	8	14	6	33
	18°C P	47.30 ± 3.30	45	2	5	5	7	4	1	3	1	1	2	3	7	4
	25°C Mono	31.60 ± 6.08	34	4	1	1	3	4	NA	NA	1	2	NA	3	1	14
	25°C P	52.50 ± 2.20	105	7	8	9	29	2	2	10	NA	2	NA	3	8	25
Total	18°C Mono	80.80 ± 2.57	223	25	15	14	33	17	16	17	14	11	8	14	6	33
	18°C P	57.20 ± 4.02	45	2	5	5	7	4	1	3	1	1	2	3	7	4
	25°C Mono	51.30 ± 9.94	34	4	1	1	3	4	NA	NA	1	2	NA	3	1	14
	25°C P	53.0 ± 2.27	105	7	8	9	29	2	2	10	NA	2	NA	3	8	25

Table S10. The individual line sample sizes when analysing the effect of sterility on remating probability in females mated to control males.

Sterile	Mean ± SE	BF1	BF11	BF12	BF14	BF3	BF4	BF7	BF8	DG2	DG3	DG4	DG7	DG8
No	0.08 ± 0.02	23	16	15	35	18	13	16	16	11	7	16	11	29
Yes	0.57 ± 0.07	4	4	6	6	5	4	4	NA	1	3	2	2	8

Table S11. The individual line sample sizes when analysing the effect of initial offspring production on the remating probability in females mated to fertile control males.

BF1	BF11	BF12	BF14	BF3	BF4	BF7	BF8	DG2	DG3	DG4	DG7	DG8
26	20	18	38	20	15	17	15	12	8	16	13	33
